# The effectiveness of albendazole against hookworm infections and the impact of bi-annual treatment on anaemia and body mass index of school children in the Kpandai district of northern Ghana

**DOI:** 10.1371/journal.pone.0294977

**Published:** 2024-03-01

**Authors:** Buhari A. Hamidu, Edward J. Tettevi, John A. Larbi, Bright K. Idun, Elias K. Asuming-Brempong, Mike Y. Osei-Atweneboana

**Affiliations:** 1 Biomedical and Public Health Research Unit, CSIR-Water Research Institute, Accra, Ghana; 2 Department of Theoretical and Applied Biology, College of Science, Kwame Nkrumah University of Science and Technology, Kumasi, Ghana; UMass Chan: University of Massachusetts Medical School, UNITED STATES

## Abstract

The impact of the Global Programme to Eliminate Lymphatic Filariasis (GPELF) (initiated in 2000 in Ghana and ran for 12 years) in mitigating soil-transmitted helminth (STH) infections in LF-endemic areas is unknown. During a 1-year hiatus which ensued between 2011 and 2012, a longitudinal study was conducted to determine GPELF effect on hookworm infections in selected communities involved in the programme since its inception, while measuring the effectiveness of biannual ALB treatments on schoolchildren living in such communities. A total of 399 school children aged 3 to 18 years were randomly selected from four communities in the Kpandai district of northern Ghana. Each presented a single stool sample at baseline, 21 days post-treatment, at the 3^rd^ and 6^th^ months, 21 days post-second intervention (i.e. following sample collection and treatment with ALB in the 6^th^ month), and in the ninth month of the study period. Haemoglobin (hb) levels were also measured at all time points using finger prick blood samples and a URIT digital test kit. Each participant submitting a sample, was treated with a single-dose ALB (400mg) at baseline and in the sixth month. Stool samples were processed by preparing duplicate Kato-Katz slides per sample, and examined by microscopy. The Body Mass Index-for-age z-scores (BAZ) of participants were assessed following the determination of BMIs at each time point by measuring their height and weight with a stadiometer and weighing scale. Overall hookworm prevalences were 25.68% (95% CI = 20.51–31.75) at baseline, 11.18% (95% CI = 7.87–15.41) 21 days post-treatment, 11.78% (95% CI = 8.38–16.11) and 6.95% (95% CI = 4.41–10.43) in the 3^rd^ and 6^th^ months, 0.91% (95% CI = 0.19–2.65) 21 days post-second intervention, and 8.46% (95% CI = 5.62–12.23) in the ninth month. Observed overall faecal egg count reduction rates (ERRs) were 94.21% (95% CI = 81.50%– 100.00%) 21 days after baseline treatment, 97.70% (95% CI = 85.08–100.00) and 96.95% (95% CI = 84.18%– 100.00%) in the 3^rd^ and 6^th^ months, 99.98% (95% CI = 86.42%– 100.00%) 21 days post-second intervention, and 17.18% (95% CI = 14.07%– 20.67%) in the 9^th^ month. Respective cure rates (CRs) were 62.35% (95% CI = 46.71–81.56%), 85.88% (95% CI = 67.32–100.00%), 87.06% (95% CI = 68.36%– 100.00%), 98.82% (95% CI = 78.83%– 100.00%), and 36.36% (95% CI = 9.91%– 93.11%). Additionally, increases in the percent frequency of ‘normal hb’ (p < 0.01) were observed across the study time points, whilst ‘normal BAZ’ cases remained high (from 94.87% to 98.87%) throughout the study period. These findings primarily indicate satisfactory effectiveness of ALB which may be maintainable in mass drug administration programmes by the modification of treatment strategies from annual to bi-annual regimes. This could minimize the likelihood of emerging poorly-responding hookworm phenotypes in Ghana. Additionally, a positive impact of bi-annual treatment on participant anaemia status is herein indicated with particular regard to the school children in our cohort.

## Introduction

Globally, approximately 1.6 billion people are infected with at least one or more of these intestinal helminths: *Ascaris lumbricoides* (roundworm), *Necator americanus*/*Ancylostoma duodenale* (hookworm), and *Trichuris trichiura* (whipworm). Most of those infected are children living in rural or resource-limited areas of low-to-middle-income countries (LMICs) [1, 2]. Up to 740 million of these infections are attributable to hookworm, and although mass chemotherapy programmes implemented by the WHO for more than a decade have helped reduce the economic burden of STHs to approximately 800,000 Disability adjusted Life Years (DALYs) as at 2015 from initial estimates of 5.19 million DALYS reported nearly 2 decades ago [3–7], much remains to be done if the WHO goal of eliminating STH infections as a Public Health challenge (i.e. where infection prevalence in current areas of endemicity is < 2%) by 2030 is to be achieved [7]. In Ghana, evidence from a national, school-based cross-sectional parasitological survey conducted in 2008 indicated hookworm infections to be highly focal, and suggested areas of highest infection to be within the northern and middle zones of the country [8]. Hookworm infections are caused through larval penetration of the human skin. The infection is usually mild but chronic, and is an important cause of iron and protein deficiency which may lead to complications during child-birth, as well as impaired physical and cognitive development in children [9].

Currently, the World Health Organisation (WHO) recommends targeted school-based annual mass drug administration (MDA) particularly of albendazole (ALB) or mebendazole (MEB, 500mg) (among others) in high-burden areas of LMICs [4]. In the year 2000, the WHO initiated the Global Programme to Eliminate Lymphatic Filariasis (GPELF) of which Ghana has been a part (through the Neglected Tropical Diseases Programme of the Ghana Health Service). Up to the commencement of this study, many communities endemic for lymphatic filariasis (LF) had received about 12 annual rounds of IVM/ALB at standard doses of 200μg/kg and 400mg respectively through community-based distributors. However, there is growing concern that sub-optimally responding hookworm phenotypes to ALB may be emerging. Findings from studies conducted in the Kintampo North Municipality of the Brong Ahafo Region (BAR) in 2007 and 2010 suggested the potential emergence of poorly-responding phenotypes among school-aged children within the municipality, the inferences of which were based on observed modest ERRs and CRs, as well as in vitro egg hatch assays (EHA) which presented with less-than-ideal respo2nses of larval isolates to ALB [10]. It has been suggested that the strategy of administering one round of a single-dose of ALB (400mg)) per annum may potentiate the emergence of these poor responders [11–13], hence the need to review the current strategy of STH preventive chemotherapy (especially in settings of heavy infections) to avoid under-dosing [14]. In this study, we assessed the effectiveness of bi-annual ALB administration on hookworm and other STHs using coprological techniques. We also determined its impact on the anaemic and nutritive statuses of schoolchildren in the Kpandai district of northern Ghana over a period of nine months, using haematological and anthropometric techniques.

## Methodology

### Ethics statement

Ethical approval for this work was obtained from the Institutional Review Board of the Council for Scientific and Industrial Research (CSIR-IRB; reference number RPN 002/CSIR-IRB/2012). Approval from chiefs, opinion leaders, and head teachers of the schools in the selected communities were also received after education on STH had been provided, and the study objectives explained. Signed or thumb-printed consent forms were collected from parents/guardians of selected pupils, or directly from pupils aged 18 years.

### Study area

The Kpandai district is located within the transitional zone of Ghana, with erratic rainfall patterns which peak in September and October; and fairly high temperatures ranging between 29°C in April and 40°C in December. The four study communities in which this work was conducted were Jagbengbendo (08.17’39.8”N; 000.07’32.1”W), Kojobone (08.29’07.7”N; 000.10’59.3”W), Takumdo (08.18’55.3”N; 000.07’53.3”W), and Wiae (08.19’21.9”N; 000.09’42.9”W) (S1 Fig). Selection of these communities was based on data obtained from the Neglected Tropical Diseases Programme (NTDP) of the Ghana Health Service, which provided information on communities in northern Ghana involved in the GPELF since its commencement in 2000. Three of the communities, Jagbengbendo, Takumdo, and Wiae, are approximately 5km apart, with Kojobone a little over 20km north of Wiae (S1 Fig). A government-managed preparatory and junior high school in each of the four study communities catered to the educational needs of the children; whilst farming was the predominant occupation among adults, followed by petty trading and fishing.

### Study design

**Fig 1:**
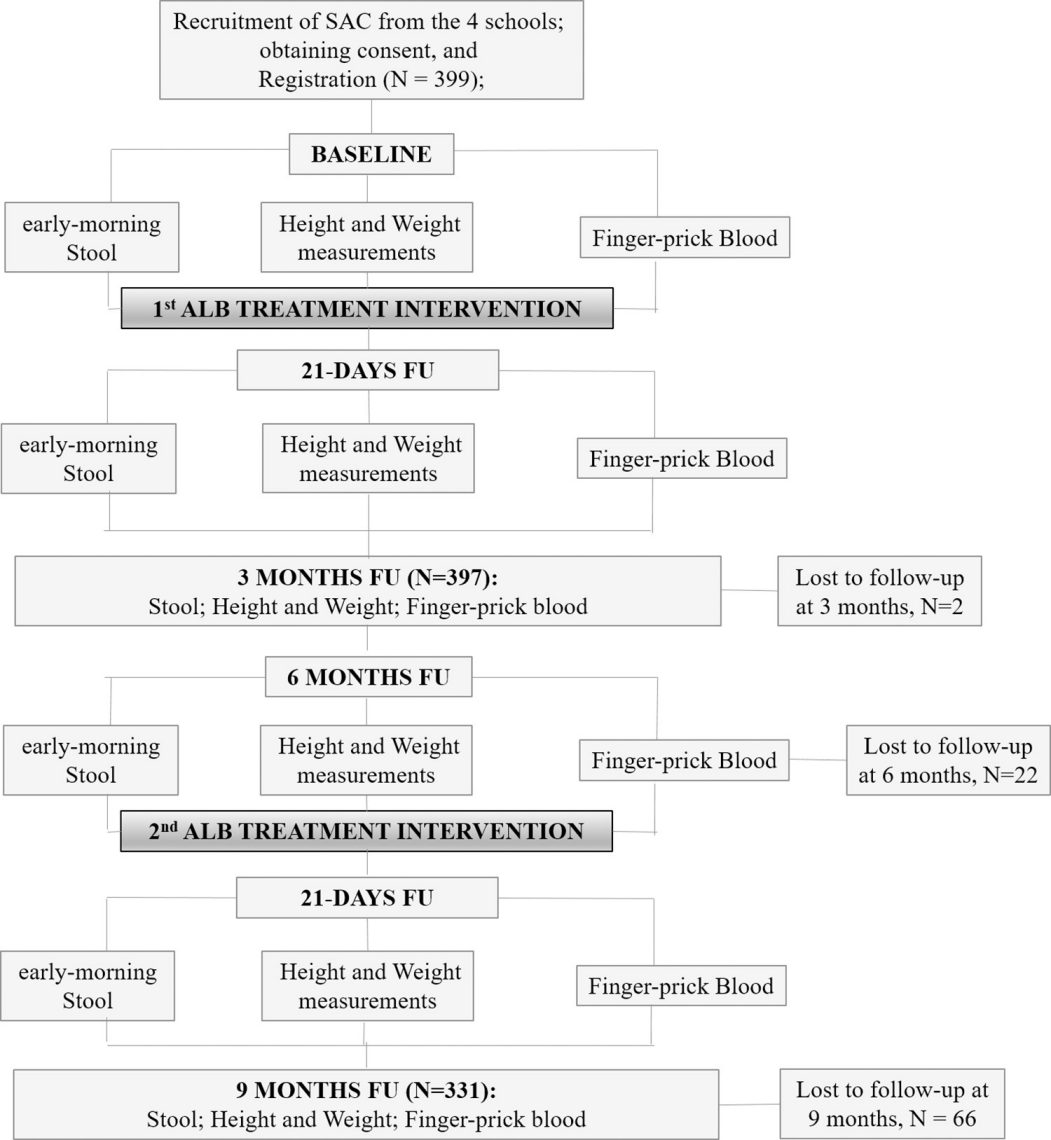
Study flow chart detailing the number of participants recruited, as well as the number assessed at each stage of the study. Also shown is the number of participants lost to follow-up at various stages; as well as the study parameters collected at each time point. FU = Follow Up.

This was a nine-month longitudinal study (conducted from mid-February to mid-November 2012) aimed at assessing the effectiveness of ALB against hookworm in selected endemic communities in the Kpandai district that had been under the GPELF since its commencement in the year 2000. Additionally, we sought to determine the health impact of a bi-annual ALB treatment regimen on pupils who fully participated in the study (Fig 1). The work involved two single-dose ALB treatment interventions 6 months apart. At the start of the study, a total of 399 pupils aged 3 to 18 years were recruited by random selection from kindergarten to class 6 (using the random number generator application software [UXAPPS, Russia]) of the selected schools in the four communities. Selected pupils provided early-morning stool samples in sterile cups (provided the day before), which were processed for microscopy. Finger-prick blood samples, as well as height and weight measurements were obtained from each subject, after which each was administered a tablet of ALB (400mg; GlaxoSmithKline Pharmaceutical Limited, India; and Zeneca, United Kingdom) under direct supervision, irrespective of infection status. Post-treatment assessment was conducted 21 days, three months (N = 397), six months (N = 375), 21 days after the second intervention with ALB, and nine months (N = 331) after baseline treatment. At each time point, finger-prick blood and stool samples, as well as height and weight measurements were obtained, processed and assessed (Fig 1).

### Study population

The representative sample size per community was determined using Cochran’s formula, n = Z^2^ [(p(1 –p))/e^2^]; whereby Z is a constant dependent on the level of acceptable α error (which in this instance is 1.96 for an α error of 0.05), p is the estimated prevalence of hookworm infections in the district (i.e. 28%, obtained from the Kpandai District Health Directorate), and e is the error, or anticipated standard deviation (i.e. 0.08 in this instance). A loss to follow-up of 20% over time was also factored into calculations, leading to a total of 120 participants per community, and an initial overall recruitment size of 480 participants. Of this number, 399 schoolchildren agreed to participate in this work–well above the calculated minimum sample size of 384 participants.

### Stool sample collection and parasitic diagnosis

Stool samples obtained from recruited participants at each time point were transferred on the day of receipt to the field laboratory in a cool box at 4°C and immediately processed for microscopy using the Kato-Katz kit with the 41.70 mg card template (Vestergaard Frandsen), as described elsewhere [15, 16]. Two slides were prepared per sample and microscopically examined within 30 minutes of preparation, for the ova of hookworm and other helminthes. For each STH species (namely, *A*. *lumbricoides*, *T*. *trichiura*, and the hookworms), the mean egg count from the two slides, was multiplied by a factor of 24 and expressed as eggs per gram (epg) of stool. Infection intensities were also categorized as light, moderate, or heavy based on WHO guidelines [17]. With regard to hookworm infections, ERRs for respective communities were calculated based on WHO guidelines for STH assessment [18], and with formulae recommended by Vercruysse and colleagues [19]. Hence, ERR and CR calculations were restricted to participants who were positive at baseline for hookworm, and who presented stool samples for assessment at all follow-up time points. Both rates were calculated for the 21 days, 3^rd^ and 6^th^ month follow-up time points using baseline data as the reference point, whilst ERR and CR for the 21 days and 3^rd^ month post-second intervention (referred to subsequently as the 9^th^ month for the sake of convenience) were calculated using the 6^th^ month follow-up time point (prior to 2^nd^ intervention) as baseline; and ERRs and CRs less than 90% were considered sufficient grounds for raising concerns about the potential emergence of poorly-responding hookworm, phenotypes to ALB [19].

### Assessment of participant anaemia status and body mass index (BMI)

Participant anaemia status was assessed at each time point, with the aid of a URIT haemoglobin (hb) test kit comprising a URIT digital Hb Meter (URIT, Guangxi Province, China) and test strips. Briefly, the test strip was inserted in the meter. Next, the thumb of the participant was pricked with a sterile lancet and the blood drop (approximately 2μL) collected in a designated groove in the meter which was absorbed by the exposed portion of the strip. The device then presented with haemoglobin counts after approximately 10 seconds. Based on outcomes and in tandem with the WHO standard criteria for anaemia, participants were categorized as either normal (hb > 10.90g/dl), mildly anaemic (10.0g/dl ≤ hb ≤ 10.9g/dl), moderately anaemic (7.0g/dl ≤ hb ≤ 9.9g/dl), or severely anaemic (hb < 7.0g/dl) [20]. Prior to analyses, these anaemia strata were re-categorized into two groups, namely the normal condition (or non-anaemic children), and the anaemic condition, which comprised all pupils with anaemia irrespective of degree of severity. Additionally, height and weight measurements of participants were taken using a stadiometer and weighing scale (RGZ-160, Jiangsu, China) and the data used to calculate the BMI for each participant. All BMIs were converted to BMI-for-Age z-scores (BAZ), and each score categorized as either low BAZ (underweight), normal BAZ (normal weight), or high BAZ (overweight/obese) based on WHO-approved stratifications. The BAZ was computed using the WHO anthropometric calculator (Anthro version 3.2.2; http://who-anthroplus.software.informer.com/1.0/) [10, 21].

### Statistical analysis

In the conceptual framework for this study, the hypothesis that semi-annual ALB treatment interventions could reduce hookworm infections in an endemic population to near-zero levels, and improve the health of schoolchildren was tested; along with associations to such *a priori* factors as gender, age, infection with other STHs, and community of residence. The null hypothesis was that, semi-annual ALB treatment would neither change hookworm infection levels in an endemic population nor improve the health of schoolchildren. All demographic and parasitological data were entered in Microsoft Office Excel version 10 (Microsoft Corporation, USA) (S2 Data), and exported to SPSS version 20 statistical software (IBM Corporation, Chicago Ill, USA) for further analysis (S1 Data). Graphs were constructed using the GraphPad Prism software version 5 (GraphPad, La Jolla CA, USA) and the MedCalc® Statistical Software version 20.218 (MedCalc Software Ltd, Ostend, Belgium). Comparisons between age, and gender groupings were conducted using the chi-square (χ^2^) test; whilst the Cochran’s Q test for related samples was used to assess variations in hookworm infection prevalences over the study period for both the pooled and community datasets. The Mann Whitney U test was employed to assess differences in hookworm infection intensities between groups within the same time point, whilst the Wilcoxon matched-pairs signed ranks test was utilised to detect variations in outcomes for same individuals two time points at a time. The associations between covariates and hookworm infection status was assessed with logistic regression analysis under the generalized estimating equation (GEE) model which ensured that, participant clustering by communities, as well as the effect of treatment over time, were accounted for. Covariates tested in the univariate model were gender, age, other STHs, and community of residence. In the multivariate model, covariates showing significant influence in the crude analyses were adjusted for, with age and gender as *a priori* confounders. Additionally, a separate model was fitted with a product term to assess the effect of treatment interactions with age, gender, and other STH infections on hookworm infection status over time. Similar univariate and multivariate models with anaemia status (normal versus anaemic as outcome variables were fitted to assess the effect of such covariates as age, gender, other STHs, hookworm infection status, and community of residence. Here as well, the effect of treatment interactions with age, gender, other STHs, and hookworm infections on anaemia status was assessed separately. In all models, each output was presented as an odds ratio (OR) with a 95% confidence interval (CI); and associations/interactions with p values < 0.05 were considered statistically significant.

## Results

### Characteristics of study participants

Of the 331 pupils who were successfully followed up on throughout the study, 98 hailed from the Jagbengbendo community, 82 from Kojobone, 84 from Takumdo, and 67 from Wiae (S2 and S3 Tables). The males constituted 46.22% (N = 153) of the 331 schoolchildren, whilst females made up 53.78% (N = 178). The ages of the participants also ranged from 3 to 18 years, with a mean age of 8.77 years (standard error of mean (SEM) = ± 0.19) (Table 1).

**Table 1:** Demographic information on study participants.

Parameter	All/Pooled [N = 331]						
	Baseline	21d	3 months	6 months		21d	9 months
Age range (min—max)	3–18	-	-	-		-	-
Mean Age (SEM)	8.77 (±0.19)	-	-	-		-	-
Age in years [n (%)]							
≤ 6	106 (32.02)	-	-	-		-	-
7–9	83 (25.08)	-	-	-		-	-
10–12	90 (27.19)	-	-	-		-	-
≥ 13	52 (15.71)	-	-	-		-	-
P-val	0.052						
Gender [n (%)]							
Males	153 (46.22)	-	-	-		-	-
Females	178 (53.78)	-	-	-		-	-
P-val	0.169						
Hookworm [n (% [95% CI])]							
^₲^infected	85 (25.68 [20.51–31.75])	37 (11.18 [7.87–15.41])	39 (11.78 [8.38–16.11])	23 (6.95 [4.41–10.43])		3 (0.91 [0.19–2.65])	28 (8.46 [5.62–12.23])
light	84	37	39	23		3	28
Moderate	1	0	0	0		0	0
^¥^P-val	**<0.001**						
Other Helminthes* [n (%[95% CI])]							
^¥^*T*. *trichiura* infected	4 (1.21 [0.33–3.09])	1 (0.30 [0.08–1.68])	2 (0.60 [0.07–2.18])	0 (0.00)		1 (0.30 [0.08–1.68])	1 (0.30 [0.08–1.68])
P-val	**<0.001**						
*H*. *nana* infected	22 (6.65 [4.17–10.06])	17.00 (5.14 [2.99–8.22])	9 (2.72 [1.24–5.16])	3 (0.91 [0.19–2.65])	0.00 (0.00)		4 (1.21 [0.33–3.09])
P-val							
Anaemia status [n (% [95% CI])]^⸸^							
Normal hb level	64 (19.30 [14.89–24.69])	-	120 (36.25 [30.06–43.35])	155 (46.83 [39.75–54.81])		-	164 (49.55 [42.25–57.74])
P-val	**<0.001**						

SEM = Standard error of mean; STH = soil-transmitted helminths; BMI = body mass index; BAZ = BMI-for-age z scores; ERR = faecal egg count reduction rate; CR = cure rate

₲ Hookworm infection intensities were categorized as ‘light’, ‘moderate’ or ‘heavy’ based on WHO guidelines (2001) for classifying infection intensities using the Kato-Katz technique.

* Other Helminthes essentially denote *Trichuris trichiura* and *Hymenolepis nana*. No participant was found positive with *A*. *lumbricoides* throughout the study.

¥ P-values for Hookworm and Other Helminthes infections were determined using the Cochran’s test for related samples. Significant values are in boldface.

**⸸** Anaemia status is a binary re-categorization of the various anaemia categories, whereby, participants with normal haemoglobin levels were grouped as ‘normal’, whilst subjects initially categorized as mildly, moderately, or severely anaemic were all grouped as ‘anaemic’.

Group comparisons over the study period were done using the Cochran’s test. Significant values are in boldface.

### Community ALB treatment history

The GPELF commenced in the year 2000 for all four study communities, and given the stipulated annual IVM/ALB MDAs, twelve rounds of IVM/ALB treatment were expected prior to the start of this work. Among the four, Kojobone and Takumdo received all twelve, whilst Jagbengendo and Wiae missed one and four rounds respectively (Table 2).

**Table 2:** Albendazole treatment histories for the study communities.

Community	ALB^1^ Treatment History				
	Year of GPELF^2^ (ALB + IVM) commencement	Rounds of treatment received prior to study commencement (ALB + IVM)	Years missed	Last GPELF treatment prior to start of study	Number of treatments administered during study (ALB only)
**Jagbengbendo**	2000	10	2009	April, 2011	2
**Kojobone**	2000	12	-	November, 2011	2
**Takumdo**	2000	12	-	September, 2011	2
**Wiae**	2000	8	2005, 2006, 2007, 2009	November, 2011	2

^1^ALB = Albendazole

^2^ GPELF = Global Programme to eliminate Lymphatic Filariasis.

### Infection measures of hookworm and other helminthes

**Fig 2:**
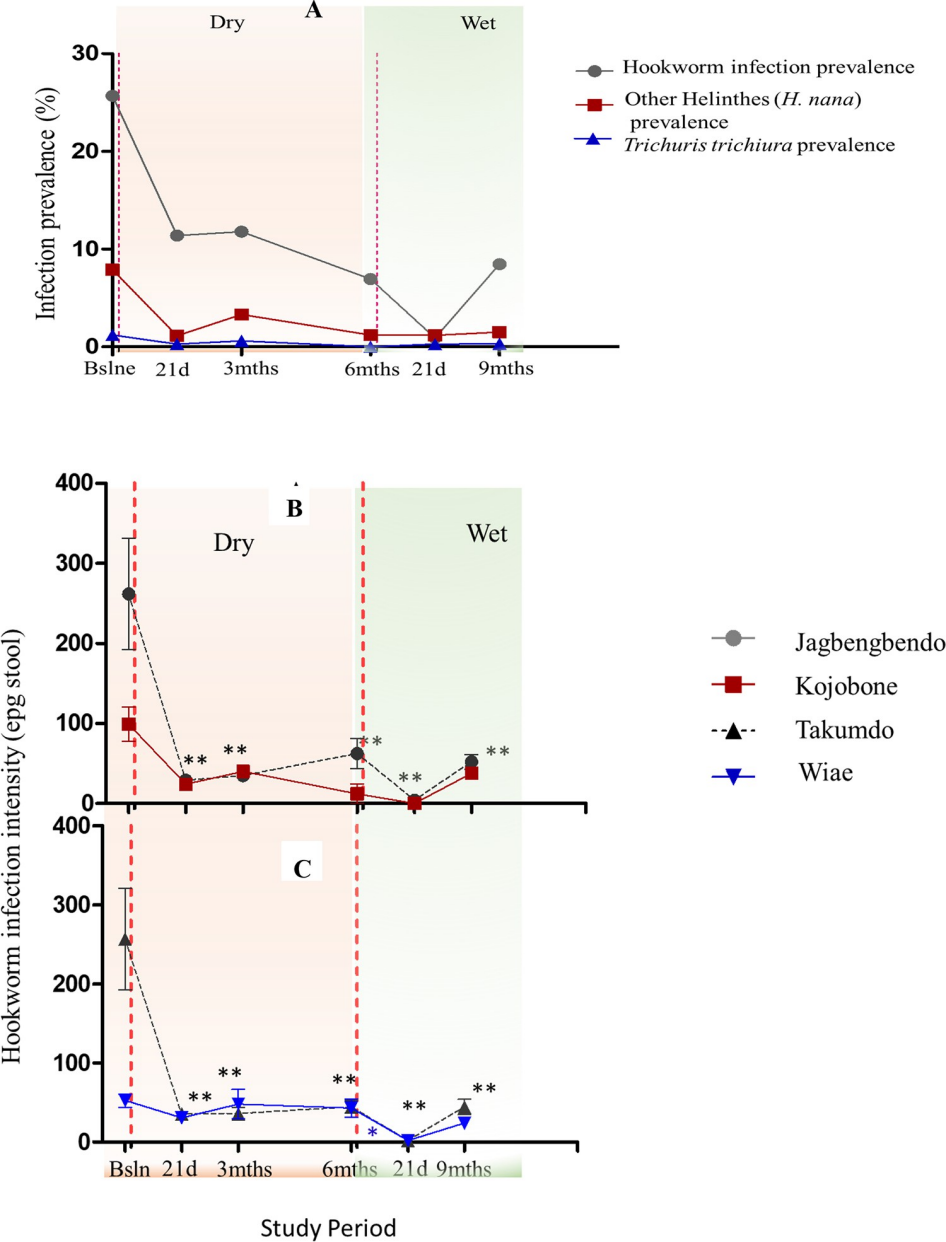
Impact of albendazole (ALB) treatment interventions on (A) the prevalences of hookworms and other Helminthes in the study cohort; and (B-C) on community-stratified hookworm infection intensities over the study period. Two ALB treatment interventions were administered to recruited school children at baseline (after parasitological screening) and in the 6^th^ month (after parameters of interest had been collected and assessed). No *A*. *lumbricoides* was observed throughout the study. Vertical, red-dashed lines demarcate treatment intervention time points.

Overall hookworm prevalence among the study participants (N = 331) at baseline was 25.68% (95% CI = 20.51–31.75). This decreased significantly to 11.18% (95% CI = 7.87–15.41) 21 days post-treatment, and increased slightly to 11.78% (95% CI = 8.38–16.11) at the 3-month time point. Prevalences at the 6^th^ month and 21 days post-2^nd^ intervention time points were 6.95% (95% CI = 4.41–10.43) and 0.91% (95% CI = 0.19–2.65) respectively. An increase to 8.46% % (95% CI = 5.62–12.23) was observed in the 9^th^ month (i.e. three months after the second ALB treatment intervention) (Table 1; Fig 2). Among the four study communities, baseline prevalences ranged from 45.92% (Jagbengbendo) to 9.76% (Kojobone) (S2 Table), with 21 days post-second-intervention prevalences (i.e. following the sixth-month assessments) approaching 0.00% for all communities. The overall infection prevalence with regard to other helminths (comprising *Trichuris trichiura*, and *Hymenolepis nana*) at baseline was 7.85%. This decreased over the study period to 1.51% in the 9^th^ month (Table 1; Fig 2). No sample was found positive for *A*. *lumbricoides* at any time point of the study.

The highest hookworm infection intensities at baseline were observed in Jagbengbendo (263.45 ± 71.22 epg) and Takumdo (286.40 ± 69.54 epg) (S2 Fig). Comparatively lower baseline intensities were observed for Kojobone (99.00 ± 22.48 epg) and Wiae (55.38 ± 9.56 epg). With the exception of Wiae, intensities declined significantly and remained low over the remaining time points. Intensities ranged between 0.00epg and 10.00epg 21 days post-second intervention for all communities (S1 Table).

### Faecal egg count reduction rates (ERRs) for hookworm infections

**Fig 3:**
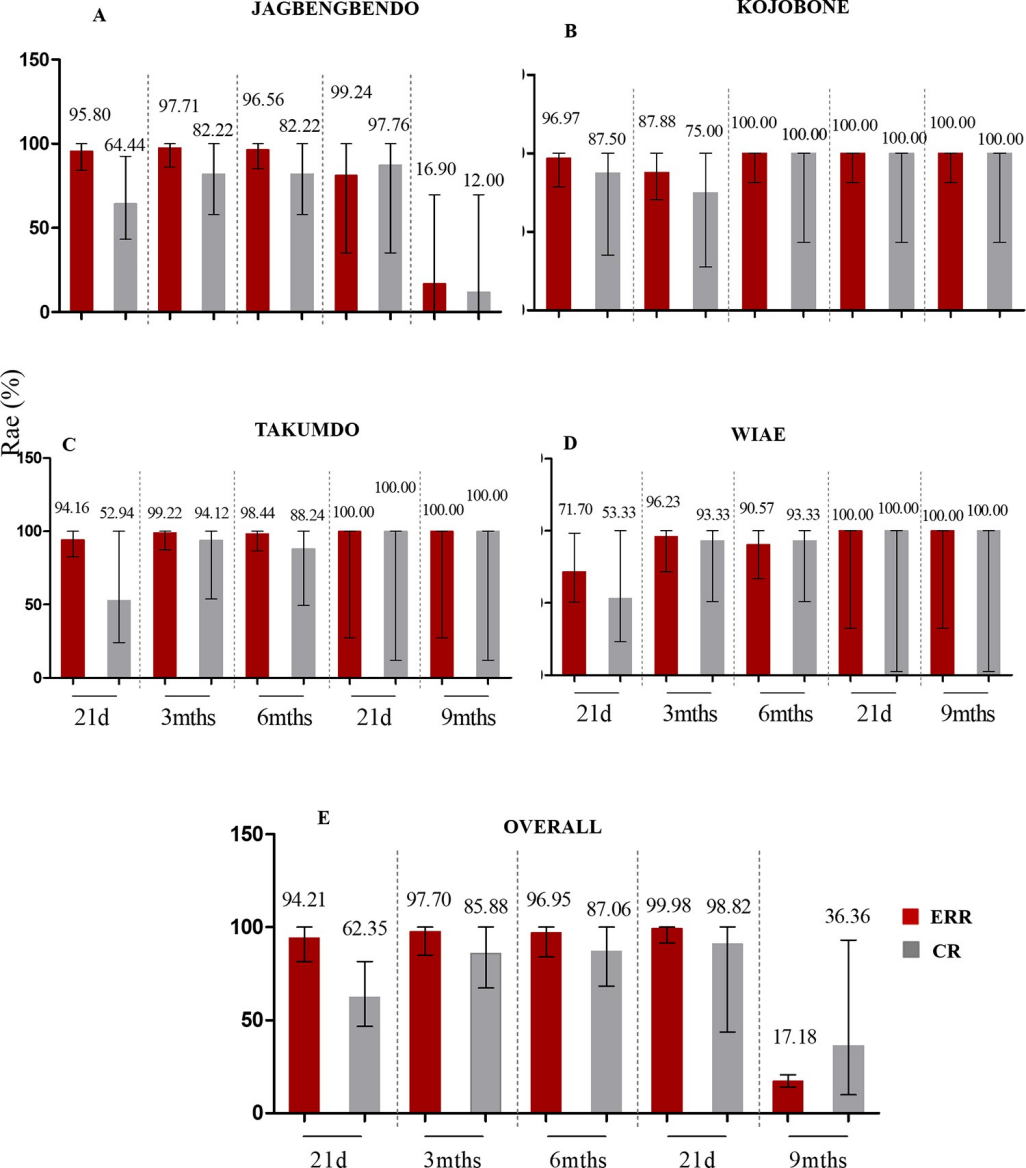
Faecal egg count reduction (ERR) and cure rates (CR) with respect to hookworm infection over time. Faecal and egg reduction rates over time for (A) Jagbengbendo, (B) Kojobone, (C) Takumdo, and (D) Wiae. (E) Overall ERR and CR for the four study communities over time. Error bars represent the 95% Confidence Intervals (CI) of prevalences.

Faecal egg count reduction rates (ERRs) were determined at all follow-up time points for a total of 85 participants from the four study communities (i.e. 44 from Jagbengbendo, 8 from Kojobone, 17 from Takumdo and 15 from Wiae). Overall ERRs ranged from 17.18% (95% CI = 14.07%– 20.67%) to 99.98% (95% CI = 86.42%– 100.00%), with the highest rate observed at the 21 days post-second-intervention time point. These ERRs were reflective of the rates observed for the individual study communities (Fig 3; S4 Table).

Additionally, overall hookworm cure rates (CRs) ranged from 36.36% (95% CI = 9.91–93.11%) to 98.82% (95% CI = 78.83%– 100.00%) over the study period, with the highest CR observed also at the 21 days post-second-intervention time point (Fig 3). Both ERR and CR calculations for the 3- and 6-month post-treatment time points were done with reference to baseline outputs, whilst ERR and CRs 21-days and 3 months (9^th^ month time point) post second intervention were determined with reference to the sixth month time point (S4 Table).

### Association of hookworm prevalence and intensity with age

**Fig 4:**
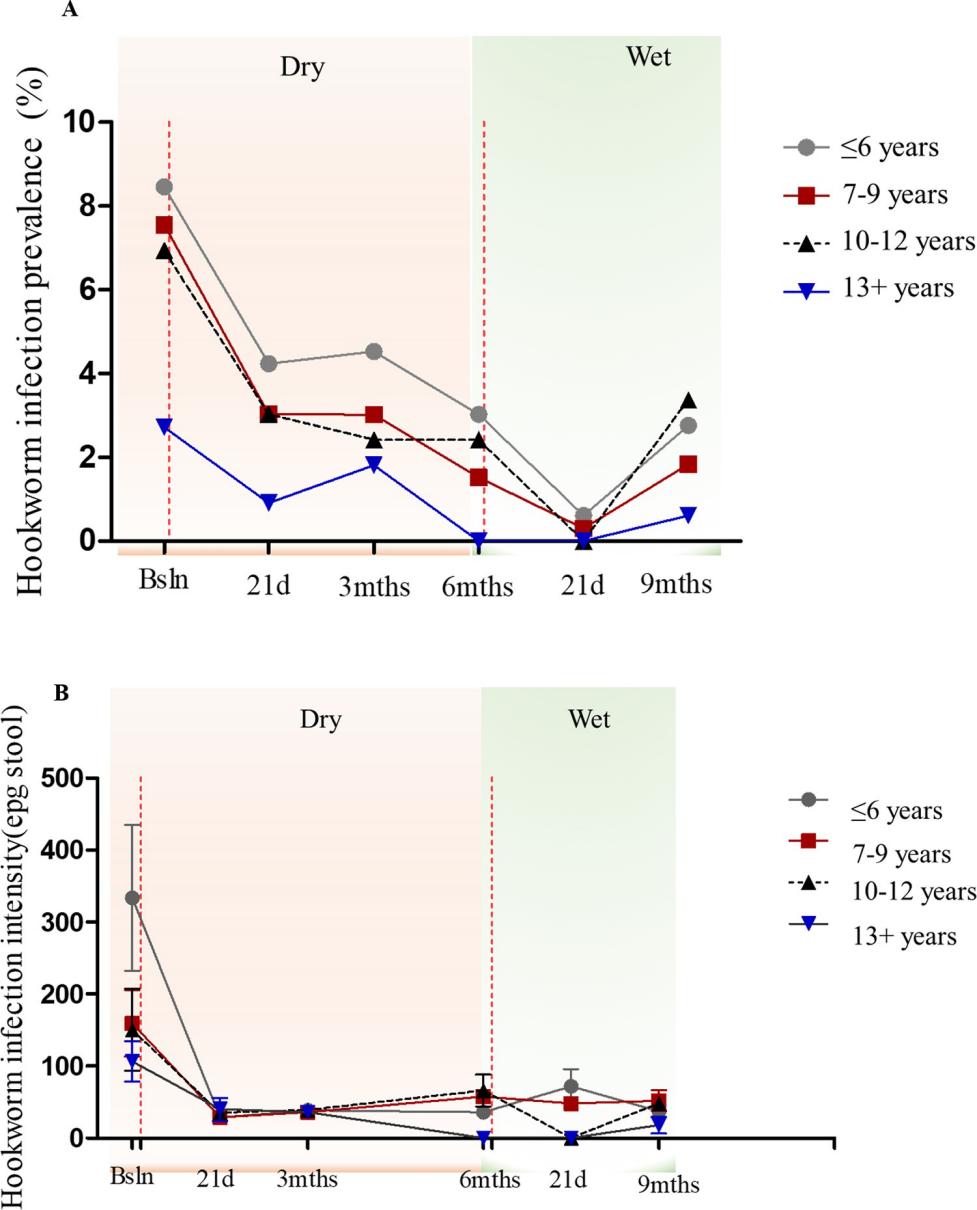
Hookworm infection distribution by age groups over the study time points. (A) Hookworm infection prevalence distribution by age groups for all study time points. (B) Hookworm infection intensity distribution by age groups for all study time points. Marker and error bars represent the arithmetic mean and standard error of mean (SEM) respectively. Red vertical dashed lines demarcate treatment intervention time points.

Baseline hookworm prevalences were highest among pupils aged 6 years or less (i.e. 8.46%), followed by pupils aged 7 to 9 years (i.e. 7.55%), SAC aged 10 to 12 years (6.95%), and for pupils aged 13 years and above (i.e. 4%). Hookworm infection prevalences for all the age groups generally declined considerably at 21days post-treatment, increased or plateaued at 3 months, and decreased further by the 6^th^ month. Hookworm prevalences for all age groups ranged between 2.00% and 0.00% 21 days post-second intervention, and increased slightly in the 9^th^ month (Fig 4A; S5 Table).

Similarly, the highest baseline hookworm infection intensity levels were realized among pupils aged 6 years or less (i.e. 333.43 ± 101.29 eggs per gramme of stool (epg)), and the lowest intensity levels recorded among SAC aged 13 years and above (i.e. 106.67±27.75 epg). Infection intensities declined significantly 21 days post-ALB treatment, and generally remained low at the remaining time points for all age groups. Thus, no significant change in intensity was observed for any of the age groups at the 21 days-post-second-intervention time point, and into the 9^th^ month (Fig 4B; S5 Table).

### Outcomes of anaemia status and BAZ assessments in pupils

**Fig 5:**
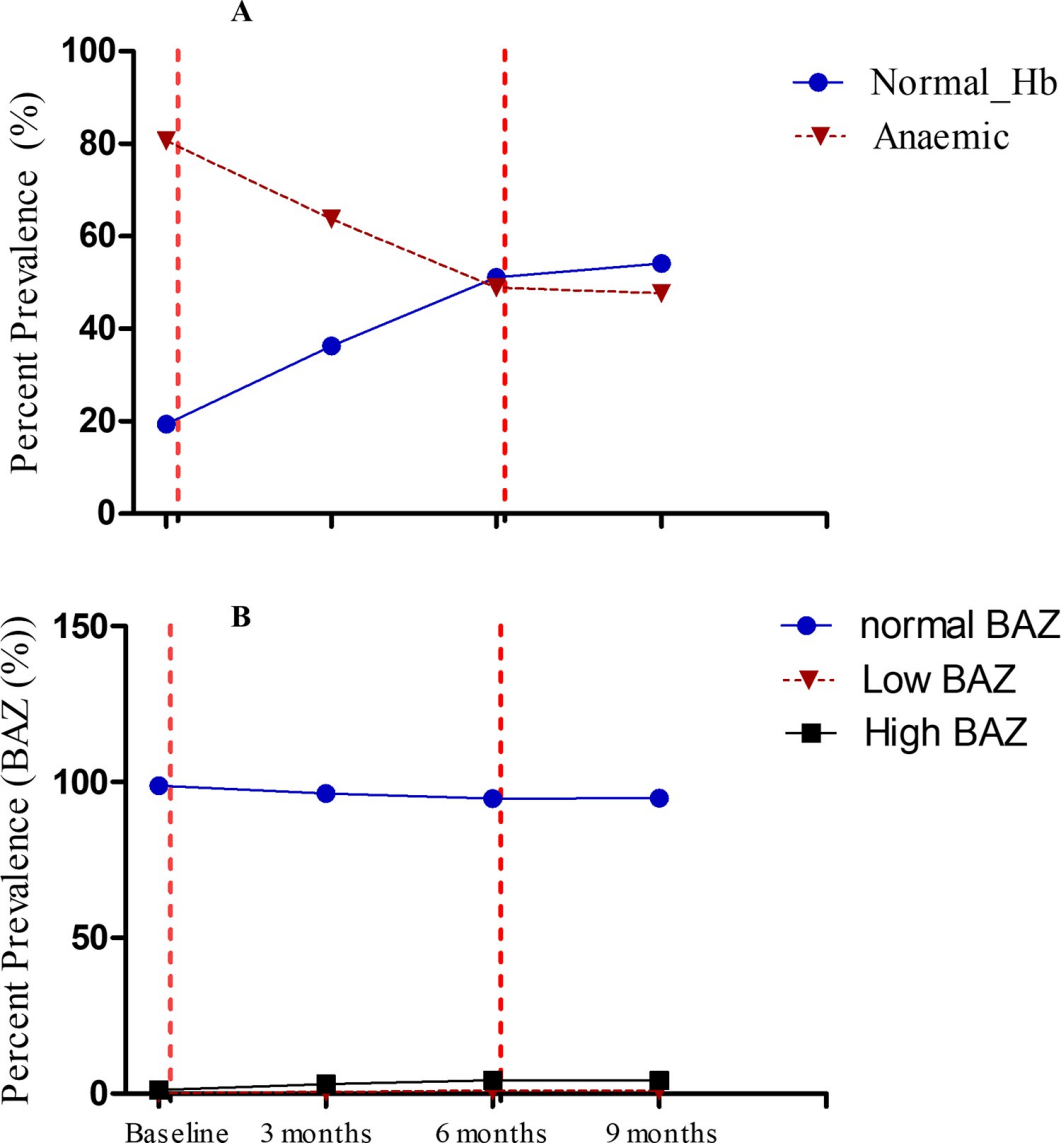
(A) Percent prevalence of participants with normal haemoglobin (hb) status, normal weight, and good health status over time. Comparisons were done using the Cochran’s test. Significant comparisons are with reference to the baseline group. ** = p-values < 0.01. (B) Percent prevalence of the BAZ status of participants over time for the various age groups. Vertical red-dashed lines demarcate ALB treatment time points.

Altogether, the prevalence of non-anaemic (normal) cases was 19.30% (95% CI = 14.89–24.69) at baseline. This improved to 36.25% (95% CI = 30.06–43.35) three months after ALB treatment; to 46.83% (95% CI = 39.75–54.81) in the sixth month; and to 49.55% (95% CI = 42.25–57.74) in the 9^th^ month (p < 0.001) (Table 1; Fig 5A). This trend was similarly observed for the four study communities. With regard to BAZ, 98.78% of SAC in the study cohort had normal weight at baseline. This decreased slightly to 96.37% 3 months after ALB treatment, and to 87.61% in the sixth month, and improved marginally in the 9^th^ month to 89.43% (Table 3; Fig 5B).

**Table 3:** Body mass index information on study participants.

Parameter	All/Pooled [N = 331]			
	Baseline	3 months	6 months	9 months
(BAZ) [n (%)]				
Low BAZ	0.00(0.00)	2.00(0.60)	3.00 (0.91)	3.00 (0.91)
Normal BAZ	326.00(98.78)	319.00(96.37)	290.00 (87.61)	296.00 (89.43)
High BAZ	4.00(1.21)	10.00(3.02)	13.00 (3.93)	13.00 (3.93)
P-val	0.686			

BAZ = BMI for Age z-scores.

### Association of hookworm infection status with *a priori* factors

Being 6 years or less presented with the highest likelihood for hookworm positivity with regard to age (cOR = 1.95; C.I. = 1.17–3.35; p = 0.011) in the univariate model, followed by the 7–9 –year (cOR = 1.85; C.I. = 1.09–3.14; p = 0.024), and the 10–12 –year (cOR = 1.84; C.I. = 1.09–3.09; p = 0.021) age groups. Also, being negative for other helminthes appeared to be protective (cOR = 0.19; C.I. = 0.11–0.36; p < 0.001), whilst residence in the Jagbengbendo community presented with a comparatively higher likelihood for hookworm positivity (cOR = 2.65; 1.68–4.19; p < 0.001) among the study communities. In the multivariate assessment, the 7–9 –year (aOR = 1.82; C.I. = 1.07–3.11; p = 0.028), and the 10-12-year (aOR = 1.77; C.I. = 1.04–3.01; p = 0.035) age groups still presented with higher odds for hookworm positivity; along with residence in the Jagbengbendo community (aOR = 2.61; C.I. = 1.62–4.20; p < 0.001). (Table 4).

**Table 4:** Associations between hookworm infection status and potential risk factors.

Parameter	Hookworm infection status (+ve or -ve)					
	cOR^§^	95% CI (LL-UL) ^∞^	Wald’s P-val	adjusted OR	95% CI (LL-UL)	Wald’s P-val
Gender						
Male	1.07	0.77–1.48	0.705	1.04	0.74–1.46	0.811
Female	1	-	-	1		
Age in years						
≤ 6	1.95	1.17–3.35	**0.011**	1.42	0.86–2.35	0.169
7–9	1.85	1.09–3.14	**0.024**	1.89	1.11–3.24	**0.019**
10–12	1.84	1.09–3.09	**0.021**	1.77	1.04–3.01	**0.035**
≥ 13	1	-	-	1	-	-
Other helminthes						
Positive	1	-	-	1	-	-
No eggs detected	0.19	0.11–0.36-	**<0.001**	0.23	0.13–0.41	**<0.001**
Community						
Jagbengbendo	2.45	1.55–3.88	**<0.001**	2.61	1.62–4.20	**<0.001**
Kojobone	0.65	0.34–1.09	0.096	0.67	0.37–1.23	0.193
Takumdo	1.05	0.63–1.73	0.86	1.05	0.62–1.79	0.844
Wiae	1	-	-	1	-	-

§ cOR = crude Odds Ratio

∞95% CI (LL–UL) = 95% confidence interval, LL = lower limit, UL = upper limit

* Other STHs = other soil transmitted helminths which represent *T*. *truchiura*, and *H*. *nana*. No participant was found positive with *A*. *lumbricoides* throughout the study. Univariate and multi-variate analyses with hookworm infection status as the outcome variable were conducted using logistic regression in the context of the generalized estimating equations (GEE) model. Significant associations are in boldface.

## Discussion

This longitudinal study was conducted to assess firstly, the effectiveness of a bi-annual treatment strategy involving single-dose ALB (400mg); and secondly, the impact of such a regime on the health of schoolchildren in selected study communities (in the Kpandai district in northern Ghana) involved in the GPELF since its commencement in 2001.

The recommended methodology for assessing anthelminthic drug efficacy according to the WHO guidelines [18] is by the ERR, the analysis of which is restricted to school children/participants who are positive at baseline for hookworm, and who provide samples at follow-up (that is, 14 to 21 days after treatment with ALB). Thus, restricting ERR assessments to the 21 days post-baseline and post-second intervention time points, it is apparent that ALB remains efficacious against hookworm infections, in accordance with current WHO guidelines [18]. Indeed, ERRs 21 days post-second intervention were higher than rates observed 21 days post-baseline treatment. Vercruysse *et al*. [13] also concluded from a large-scale trial involving 7 countries that ALB efficacy remained satisfactory; as did Diawara *et al*. [22], following a multi-centre study involving Haiti, Kenya, and Panama. For both studies, follow-up activities and ERR assessments were restricted maximally to 21 days after treatment.

Outcomes from this work also indicate a positive impact of a second intervention annually in maintaining low and driving down further, prevalences of hookworm infections. These were apparent in the higher ERRs and lower hookworm prevalences observed especially 21 days post-second intervention. A 3-year study conducted by Pion and colleagues in a village in the Democratic Republic of Congo in 2012 [23] assessing the effect of semi-annual community-wide treatments with ALB on LF and STH showed significant effectiveness of ALB on hookworm after two treatments 6 months apart, with up to a 100.00% reduction in prevalence among children up to 14 years of age. Also, a 2016 assessment by Adriko and team [24] of a Ugandan national deworming programme showed overall/country-wide STH prevalence to reduce from 60.0% in 2002 to 8.8% at the time of assessment. Contrarily, outcomes from studies evaluating annual ALB MDA programmes indicate a reduced effectiveness in the capacities of such programmes in maintaining reduced STH prevalence levels observed upon treatment. The evaluation of a 7-year long MDA programme in Burundi by Ortu and colleagues [25] is a case in point. Similarly, Brooker and colleagues [26] showed through mathematical models that the highest proportion of participants in a prospective trial to remain infected with hookworm were those receiving annual doses of ALB as compared to those receiving bi-annual treatment.

Worth noting was the reduced overall ERR and CR observed in the 9^th^ month (3 months post-2^nd^ intervention), which appeared to be influenced by the low ERR and CR observed for Jagbengbendo (also in the 9^th^ month). Given the proven effectiveness of ALB 21 days post-2^nd^ intervention, it became apparent that the environmental/hygienic conditions of the study communities at the time this work was conducted may have been major contributors. Observations by our field staff indicated Jagbengbendo to be the least hygienic among the 4 study communities whilst the converse was observed for Kojobone. Also, multivariate analyses outcomes indicated that being resident in Jagbengbendo presented with the highest odds for hookworm positivity, whilst residence in Kojobone seemed protective. The impact of prevailing hygienic/environmental conditions on STH proliferation became more apparent when the number of annual rounds of IVM/ALB MDAs received by our study communities since the start of the GPELF programme were considered. It was anticipated that hookworm prevalences to be realized in this study would be indicative of near-elimination, given that prior to the start of this work Jagbengbendo had received 11 annual rounds of mass chemotherapy (IVM and ALB); Wiae 8 annual rounds; and Kojobone and Takumdo, 12 annual rounds each. Nevertheless, we found Jagbengbendo to have the highest baseline hookworm prevalence rate, whilst baseline hookworm prevalences were observed for Kojobone and Takumdo even though no round was missed. Indeed, studies recently conducted (2019; unpublished data) in these communities still showed hookworm infection prevalences to be as high as almost 19.00%. Knopp and colleagues [27] reported baseline hookworm prevalences of up to 18.50% in a study involving school children hailing from two communities in Zanzibar that had been involved in the GPELF for at least a decade. Vercruysse and colleagues [13] indicated the pre-intervention hookworm prevalences of trial sites in Cambodia, Cameroon, India, and Tanzania to be 12.38%, 9.42%, 2.89%, and 68.56% respectively despite at least 7 prior years of large-scale anthelminthic treatment (LSAT) with ALB or MEB. Given these instances, it is quite apparent that, chemotherapy alone may not prove entirely adequate in driving down prevalence levels to zero, particularly where conducive environmental conditions prevail for continued STH transmission [27, 28]. The time gap between last GPELF and study commencement affirm this, with Jagbengbendo presenting with the longest time gap of 10 months prior to study commencement (which presents enough time for re-infections if conducive conditions and behaviours persist), as compared to Kojobone and Wiae. In essence, the feasibility of elimination may be more likely if other key contributing factors including the practice of good hygiene, potable water availability, as well as the improvement of community general sanitations (as encapsulated in the Water, Sanitation, and Hygiene (WASH) programme), are tackled concurrently [29, 30].

We observed considerable improvement over time (following our baseline and 6-month ALB treatment interventions) in the percent frequency of SAC with normal hb, while anaemia prevalence reduced concurrently over the study period. We also observed through our models, a significant, protective effect of hookworm negativity on anaemia status (S6 to S8 Tables). Muhangi *et al*. [31] indicated increasing hookworm intensity to be associated with lower haemoglobin levels and vice versa. Similarly, Djuardi and colleagues [32] from investigations among PSAC in Nangapanda sub-district Indonesia, indicated mild anaemia to be associated with mild STH (particularly hookworm) infection. Additionally, Molla and team [33] in South Ethiopia found hookworm infections to be highly correlated with anaemia. These findings are important as they further confirm trends observed in this study, and are also in keeping with the well-documented impact of STH infections, especially hookworm, on participant haemoglobin levels [9, 34, 35]. Humphries *et al*. [10], also reported a high prevalence of anaemia among PSAC in the Kintampo North Municipality (KNM) of Ghana. However, the impact of ALB on improving the anaemia status of PSAC could not be clearly determined due to the confounding effect of malaria which was also highly prevalent among study subjects. Interestingly, the percent frequency of participants with normal BAZ was high at baseline and varied insignificantly throughout the study. This could be a positive outcome of the GPELF, given the number of years of programme implementation in our study communities, which likely impacted positively on the health of SAC involved in the study. The impact of PH policies by the Government of Ghana regarding antenatal and post-natal care for children up to 60 months old cannot be ruled out. Certain reviews [35–38] also reported little change in BMI, including a study in Indonesia by Wiria and colleagues [39] who in a 21-month randomized controlled study, observed no significant change in BMI among study participants despite ALB chemotherapy administration every 3 months.

Infection of endemic populations by helminths has been shown extensively to be age-associated, with pre-school aged children (PSAC) and SAC being the most vulnerable groups [13, 39–41]. This is in tandem with our findings, whereby being aged 6 years or less presented with the highest odds of being hookworm positive, followed by the 7-9-year and the 10-12-year age groups concurrently. Indeed, these two age groups remained strongly associated with hookworm positivity after adjusting for confounders. Similar observations were made with regard to hookworm infection prevalence and intensity. It is worth noting that robustness of participant immune response is important for maintained resistance to infection/re-infection [42], and for the optimal efficacy of many therapeutic agents [13]. Epidemiological studies on other helminths like the *Schistosoma sp*. have shown the development of such robust immune systems to be age-dependent [42–44]. Whether this applies to hookworm remains unclear, although for this study the lowest prevalences and intensities were observed among participants aged 13 years and above.

This study presented with important limitations. The first was with regard to the design, which did not span a full season cycle (12 months), nor incorporate a head-to-head comparison with a community where a biannual ALB MDA programme was being executed. Budgetary constraints were a main cause in the observed design limitations. The second was with regard to the distribution of participants by age groups which showed significant variation particularly in Jagbengbendo and Wiae. This (despite the random selection at recruitment and registration) was due to the higher compliance among schoolchildren aged 12 years and below. Many of the recruited pupils aged 13 years and above did not present sample at a particular time point, and had to be excluded from analyses. The reduced participation of such SAC may have been due to the frequency at which samples were required in this work, coupled with the cultural influences and embarrassments associated with stool among teenagers in such settings. The distribution variations may have influenced likelihood or odds analyses in this work. It is however worth noting that this ‘skewness’ in age-group sizes almost disappears when community-level stratification is removed. A third limitation was the final number of participants who were included in ERR analyses. Out of a total of 399 recruited participants, only 85 who were positive at baseline and provided stool samples at all subsequent time points could be assessed. The high ERRs observed among such participants underscore the need for increased efforts by field staff of MDA programmes to ensure compliance of indigenes in affected areas in drug uptake for programme effectiveness. This need is more apparent when the 85 participants are further stratified on the basis of study communities.

Essentially, we provide evidence indicative of satisfactory ALB (400mg) effectiveness, which is maintainable with a bi-annual strategy (assuming optimal participant compliance and the concurrent implementation of WASH strategies); as well as a positive effect of ALB treatment on participant anaemia status. We also highlight the associations of gender, age, and hygiene, on hookworm infection probability. Finally, we show here that hookworm distribution is independent of proximity, and stress the need for reconsiderations by stakeholders for the modification of the current ALB de-worming programme in Ghana from an annual to a bi-annual treatment regimen.

## Supporting information

S1 Table Infection intensities of the 85 participants stratified by study community at all study time points. (PDF)

S2 Table Demographics of study cohort by study communities. (PDF)

S3 Table Demographics of study cohort by study communities. (PDF)

S4 Table Determination of egg reduction rates (ERR) and cure rates (CR) using different reference points of chemotherapy. (PDF)

S5 Table Association of risk factor-treatment interactions over time with hookworm infection status. (PDF)

S6 Table Associations between anaemia status and potential risk factors. (PDF)

S7 Table Association of risk factor-treatment interactions over time with anaemia status. (PDF)

S8 Table Association of risk factor-treatment interactions over time with anaemia status. (PDF)

S1 Fig A map of the Kpandai district indicating the locations of the study communities. Map was developed by Elias K. Asuming-Brempong using the QGIS Girona version 3.0.3 (Boston, MA., USA). Shape files of the regions of Ghana were obtained online (URL: https://github.com/tierney/gis-sandbox/tree/master/data/GIS-Ghana/ghana.shapefiles). Also, GPS data were obtained from the field, using appropriate devices, and exported to Microsoft Office Excel version 2013, where conversions were made. The document was then exported to the QGIS Girona version 3.0.3 software as a delimited text file.
(PDF)

S2 Fig Effect of Albendazole (ALB) treatment interventions on hookworm infection prevalences in the study communities over the study period. Two ALB treatment interventions were administered to recruited SAC at baseline (after parasitological screening) and in the 6^th^ month (after parameters of interest had been collected, and parasitological assessments done). Vertical, red-dashed lines demarcate treatment intervention time points.
(PDF)

S1 Data Dataset used in preparing article in SPSS format. (SAV)

S2 Data Dataset used in preparing article in MS Office Excel format. (XLSX)
