# Supplementary material for: The effectiveness of albendazole against hookworm infections and the impact of bi-annual treatment on anaemia and body mass index of school children in the Kpandai district of northern Ghana
Source: PLoS One. 2024 Mar 1;19(3):e0294977. doi: 10.1371/journal.pone.0294977 (PMC10906822; doi:10.1371/journal.pone.0294977)
Supplement: S1 Table — (PDF) [file pone.0294977.s001.pdf]

S1 Table: Infection intensities of the 85 participants stratified by study community at all study time points.

	**Baseline**	**21 days**	**3 months**	**6 months**†	**21 days**	**9 months**
Pooled						
N	85	85	85	85	85	85
Mean (±SEM)	217.50 ± 42.23	13.42 ± 2.07	5.10 ± 1.46	5.70 ± 1.92	1.20 ± 1.20	4.50 ± 2.14
Jagbengbendo						
N	45	45	45	45	45	45
Mean (±SEM)	263.45 ± 71.22	11.40 ± 2.62	6.00 ± 2.08	8.73 ± 3.23	2.23 ± 2.23	8.20 ± 3.82
Kojobone						
N	8	8	8	8	8	8
Mean (±SEM)	99.00 ± 22.48	3.00 ± 3.00	12.00 ± 7.86	0.00	0.00	0.00
Takumdo						
N	17	17	17	17	17	17
Mean (±SEM)	286.40 ± 69.54	23.00 ± 5.94	1.60 ± 1.60	4.80 ± 3.47	0.00	0.00
Wiae						
N	15	15	15	15	15	15
Mean (±SEM)	55.38 ± 9.56	14.40 ± 4.57	1.85 ± 1.85	0.00	0.00	0.00

† denotes the time of the second intervention, which occurred in the sixth month after participants had provided their stool, urine, and blood samples.
