# Supplementary material for: The effectiveness of albendazole against hookworm infections and the impact of bi-annual treatment on anaemia and body mass index of school children in the Kpandai district of northern Ghana
Source: PLoS One. 2024 Mar 1;19(3):e0294977. doi: 10.1371/journal.pone.0294977 (PMC10906822; doi:10.1371/journal.pone.0294977)
Supplement: S2 Table — (PDF) [file pone.0294977.s002.pdf]

S2 Table: Demographics of study cohort by study communities

Parameter	Jagbengbendo [N = 98]				Kojobone [N = 82]				Takumdo [N = 84]				Wiae [N = 67]			
	**Baseline**	**3 months**	**6 months**	**9 months**	**Baseline**	**3 months**	**6 months**	**9 months**	**Baseline**	**3 months**	**6 months**	**9 months**	**Baseline**	**3 months**	**6 months**	**9 months**
Age range (min - max)	3 - 18	-	-	-	3 - 15	-	-	-	4 - 15	-	-	-	3 - 14	-	-	-
Mean Age (±SEM)	8.19 (±0.40)	-	-	-	10.05 (±0.36)	-	-	-	9.37 (±0.31)	-	-	-	7.31 (±0.34)	-	-	-
Age in years [n (%)]**≤ 6**	46 (46.94)	-	-	-	14 (17.07)	-	-	-	18 (21.43)	-	-	-	28 (41.79)	-	-	-
**7-9**	15 (15.31)	-	-	-	17 (20.73)	-	-	-	26 (30.95)	-	-	-	25 (37.31)	-	-	-
**10-12**	25 (25.51)	-	-	-	26 (31.71)	-	-	-	29 (34.52)	-	-	-	10 (14.93)	-	-	-
**≥ 13**	12 (12.24)	-	-	-	25 (30.49)	-	-	-	11 (13.10)	-	-	-	4 (5.97)	-	-	-
**P-val**	**<0.001**				0.163				**0.024**				**<0.001**			
Gender, [n, (%)]																
Males	48 (48.98)	-	-	-	38 (46.34)	-	-	-	33 (39.29)	-	-	-	34 (50.75)	-	-	-
Females	50 (5102)	-	-	-	44 (53.66)	-	-	-	51 (60.71)	-	-	-	33 (49.24)	-	-	-
**P-val**	0.84				0.508				0.05				0.903			
Hookworm [n, (%)]																

infected[Ǥ]	45 (45.92)	22 (22.45)	10 (10.20)	12 (12.24)	8 (9.76)	6 (7.32)	1 (1.22)	7 (8.54)	17 (20.24)	6 (7.14)	7 (8.33)	7 (8.33)	15 (22.39)	5 (7.46)	5 (7.46)	2 (2.99)
Light	44	22	10	12	8	6	1	7	17	6	7	7	15	5	5	2
Moderate	1	0	0	0	0	0	0	0	0	0	0	0	0	0	0	0
**P-val**[¥]	**<0.001**				0.142				0.057				**0.002**			
Community Hookworm FECRR (%)	*n*[†] = 44				*n* = 8				*n* = 17				*n* = 15			
	-	97.72	96.69	96.89	-	87.88	100.00	100.00	-	99.45	98.35	100.00	-	96.97	90.91	100.00
Community Hookworm CR (%)	-	81.82	81.82	86.36	-	75.00	100.00	100.00	-	94.12	88.24	100.00	-	93.33	93.33	100.00
Other Helminths* [n, (%)]																
infected	12 (12.24)	6 (6.12)	2 (2.04)	3 (3.06)	2 (2.44)	2 (2.44)	0 (0.00)	0 (1.21)	6 (7.14)	2 (2.38)	1 (1.19)	1 (1.19)	6 (8.95)	1 (1.49)	1 (1.49)	0 (0.00)
**P-val**	**0.01**				0.261				0.079				**0.035**			

SEM = Standard error of mean; STH = soil-transmitted helminths; BMI = body mass index; FECRR = faecal egg count reduction rate; CR = cure rate.

***** Other Helminthes denote *Trichuris trichiura*, and *Hymenolepis nana*. No participant was found positive with *A. lumbricoides* throughout the study.

Ǥ Hookworm infection intensities were categorized as ‘light’, ‘moderate’ or ‘heavy’ based on WHO guidelines (2001) for classifying infection intensities using the Kato-Katz technique.

¥ P-values for Hookworm and Other STH infections were determined using the Cochran’s test for related samples. Significant values are in boldface.

§ The BMI-for-Age z scores is the re-categorization of BMI data for all participants.

† *n* denotes the number of participants who were positive for hookworm infection at baseline, and who provided a stool sample at all of the follow-up time points. FECR calculations were done using *n*. Group comparisons were done using the χ^2 test. Significant values are in boldface.
