# Supplementary material for: The effectiveness of albendazole against hookworm infections and the impact of bi-annual treatment on anaemia and body mass index of school children in the Kpandai district of northern Ghana
Source: PLoS One. 2024 Mar 1;19(3):e0294977. doi: 10.1371/journal.pone.0294977 (PMC10906822; doi:10.1371/journal.pone.0294977)
Supplement: S3 Table — (PDF) [file pone.0294977.s003.pdf]

S3 Table: Demographics of study cohort by study communities

**Parameter**	**Jagbengbendo [N = 98]**				**Kojobone [N = 82]**				**Takumdo [N = 84]**				**Wiae [N = 67]**			
	Baseline	3 months	6 months	9 months	Baseline	3 months	6 months	9 months	Baseline	3 months	6 months	9 months	Baseline	3 months	6 months	9 months
§BMI for Age Z scores [n, (%)]																
Normal BAZ	30 (30.61)	24 (24.49)	26 (26.53)	31 (31.63)	22 (26.83)	34 (41.46)	16 (19.51)	20 (24.39)	5 (5.95)	6 (7.14)	7 (8.33)	17 (20.24)	14 (20.89)	15 (22.39)	10 (14.93)	14 (20.89)
High BAZ	68 (69.39)	74 (75.51)	72 (73.47)	67 (68.37)	60 (73.17)	46 (56.09)	66 (80.49)	62 (75.61)	79 (94.05)	78 (92.86)	77 (91.67)	67 (79.76)	53 (79.10)	52 (77.61)	57 (85.07)	53 (79.10)
P-val	0.594				**0.0033**				**0.0029**				0.584			

SEM = Standard error of mean; STH = soil-transmitted helminths; BMI = body mass index; FECRR = faecal egg count reduction rate; CR = cure rate.

***** Other Helminthes denote *Trichuris trichiura*, and *Hymenolepis nana*. No participant was found positive with *A. lumbricoides* throughout the study.

₲ Hookworm infection intensities were categorized as ‘light’, ‘moderate’ or ‘heavy’ based on WHO guidelines (2001) for classifying infection intensities using the Kato-Katz technique.

¥ P-values for Hookworm and Other STH infections were determined using the Cochran’s test for related samples. Significant values are in boldface.

§ The BMI-for-Age z scores is the re-categorization of BMI data for all participants.

† *n* denotes the number of participants who were positive for hookworm infection at baseline, and who provided a stool sample at all of the follow-up time points. FECR calculations were done using *n*. Group comparisons were done using the χ^2 test. Significant values are in boldface.
