# Supplementary material for: The effectiveness of albendazole against hookworm infections and the impact of bi-annual treatment on anaemia and body mass index of school children in the Kpandai district of northern Ghana
Source: PLoS One. 2024 Mar 1;19(3):e0294977. doi: 10.1371/journal.pone.0294977 (PMC10906822; doi:10.1371/journal.pone.0294977)
Supplement: S4 Table — (PDF) [file pone.0294977.s004.pdf]

S4 Table: Determination of egg reduction rates (ERR) and cure rates (CR) using different reference points of chemotherapy

	**Baseline**	**21 days**	**3 months**	**6 months**	**21 days**	**9 months**[₲]
N	85	85	85	85	85	85
ERR [ref: Baseline]						
ERR (95% CI)	-	94.21%<br>(81.50 – 100.00)	97.70%<br>(85.08–100.00)	96.95%<br>(84.18–100.00)	99.98%<br>(86.42–100.00)	97.48%<br>(84.63–100.00)
ERR[†] [ref1: Baseline; ref2: 6 months]						
ERR[†] (95% CI)	-	94.21%<br>(81.50 – 100.00)	97.70%<br>(85.08–100.00)	96.95%<br>(84.18–100.00)	99.21%<br>(91.62-100.00)	17.18%<br>(14.07-20.67)
CR [ref: Baseline]						
CR (95% CI)	-	62.35%<br>(46.71–81.56)	85.88<br>(67.32–100.00)	87.06%<br>(68.36–100.00)	98.82%<br>(78.83–100.00)	91.76%<br>(72.54–100.00)
CR[†] [ref1: Baseline; ref2: 6 months]						
CR[†] (95% CI)		62.35%<br>(46.71–81.56)	85.88<br>(67.32–100.00)	87.06%<br>(68.36–100.00)	90.91<br>(43.59-100.00)	36.36<br>(9.91-93.11)

Ref = reference point; ref1 = first reference point which is the main baseline of the study during which ALB chemotherapy was administered following initial sample collection, processing and assessments; ref2 = second reference point which is in the 6th month time point (from main baseline) during which ALB chemotherapy was again administered following sample collection, processing, and assessments.

ERR = Egg reduction rate determined for all time points using the main baseline of the study as the only reference point; ERR[†] = Egg reduction rate determined using the study baseline (for 21 days, 3 months and 6 months post-baseline chemotherapy), and the sixth month time point (for 21 days, and 3 months post-6th month chemotherapy (also the 9th month)) as the reference points. CR = Cure rate determined for all time points using the main baseline of the study as the only reference point; **CR**[†] = Cure rate determined using the study baseline (for 21 days, 3 months and 6 months post-baseline chemotherapy), and the sixth month time point (for 21 days, and 3 months post-6th month chemotherapy (also the 9th month)) as the reference points.
