# Supplementary material for: The effectiveness of albendazole against hookworm infections and the impact of bi-annual treatment on anaemia and body mass index of school children in the Kpandai district of northern Ghana
Source: PLoS One. 2024 Mar 1;19(3):e0294977. doi: 10.1371/journal.pone.0294977 (PMC10906822; doi:10.1371/journal.pone.0294977)
Supplement: S5 Table — (PDF) [file pone.0294977.s005.pdf]

S5 Table: Associations of risk factor-treatment interactions over time with hookworm infection status

**Interacting Parameters**	**Hookworm infection status (+ve or -ve)**					
	cOR§	95% CI (LL-UL)	Wald's P-val	adjusted OR	95% CI (LL-UL)	Wald's P-val
Treatment * Gender						
Male * Baseline	4.11	2.18 - 7.77	**<0.001**	1.19	0.72 - 1.99	0.486
Female * Baseline	3.46	1.85 - 6.47	**<0.001**	1	-	-
Male * 3 months	1.45	0.70 - 2.98	0.314	1.02	0.5 0 - 2.06	0.968
Female * 3 months	1.45	0.72 - 2.91	0.29	1	-	-
Male * 6 months	0.84	0.38 - 1.89	0.677	1.07	0.44 - 2.59	0.886
Female * 6 months	0.79	0.37 - 1.68	0.53	1	-	-
Male * 9 months	1.01	0.46 - 2.19	0.98	1.09	0.47 - 2.53	0.848
Female * 9 months	1	-	-	1	-	-
Treatment * Age						
≤ 6 * Baseline	8.97	2.05 - 39.34	**0.004**	0.19	0.01 - 3.42	0.262
7-9 * Baseline	10.78	2.43 - 47.77	**0.002**	0.25	0.01 - 4.18	0.331
10-12 * Baseline	8.58	1.93 - 38.10	**0.005**	0.19	0.01 - 3.33	0.256
≥ 13 * Baseline	5.23	1.01 - 27.06	**0.048**	0.12	0.006 - 2.23	0.15
≤ 6 * 3 months	4.12	0.91 - 18.75	0.067	0.44	0.02 - 8.23	0.58
7-9 * 3 months	3.43	0.72 - 16.30	0.122	0.37	0.02 - 6.85	0.505
10-12 * 3 months	2.44	0.49 - 11.95	0.27	0.25	0.01 - 4.93	0.363
≥ 13 * 3 months	3.26	0.59 - 17.88	0.17	0.31	0.02 - 6.23	0.446
≤ 6 * 6 months	2.6	0.55 - 12.35	0.228	0.39	0.01 - 11.10	0.587
7-9 * 6 months	1.6	0.29 - 8.58	0.58	0.30	0.01 - 10.29	0.506
10-12 * 6 months	2.44	0.49 - 11.95	0.27	0.45	0.01 - 14.72	0.657
≥ 13 * 6 months	9.81^-12	0 - infinity	-	1.84^-12	0 - infinity	-
≤ 6 * 9 months	2.32	0.48 - 11.15	0.29	1.69	0.33 - 8.58	0.526
7-9 * 9 months	1.95	0.38 - 10.04	0.425	1.46	0.28 - 7.70	0.659
10-12 * 9 months	3.48	0.74 - 16.36	0.11	3.48	0.73 - 16.24	0.118
≥ 13 * 9 months	1	-	-	1	-	-

§ cOR = crude Odds Ratio; ∞95% CI (LL – UL) = 95% confidence interval, LL = lower limit, UL = upper limit; ⸸Other STHs = other soil transmitted helminths which represent *T. truchiura*, and *H. nana*. No participant was found positive with *A. lumbricoides* throughout the study. Univariate and multi-variate analyses of the effect of treatment-risk factor interactions over time with hookworm infection status (the outcome variable) were conducted using logistic regression in the context of the generalized estimating equations (GEE) model. Significant associations are in boldface.
