# Supplementary material for: The effectiveness of albendazole against hookworm infections and the impact of bi-annual treatment on anaemia and body mass index of school children in the Kpandai district of northern Ghana
Source: PLoS One. 2024 Mar 1;19(3):e0294977. doi: 10.1371/journal.pone.0294977 (PMC10906822; doi:10.1371/journal.pone.0294977)
Supplement: S6 Table — (PDF) [file pone.0294977.s006.pdf]

S6 Table: Associations between anaemia status and potential risk factors

Parameter	Anaemia status (anaemic vs normal)					
	**cOR**	**95% CI (LL-UL)**	**Wald's P-val**	**adjusted OR**	**95% CI (LL-UL)**	**Wald's P-val**
Gender						
Male	0.85a	0.64 - 1.14	0.289	-	-	-
Female	1	-	-	-	-	-
Age in years						
≤ 6	0.24	0.15 - 0.39	**<0.001**	5.19	2.98 - 9.08	**<0.001**
7-9	0.66	0.42 – 1.05	**0.082**	1.67	0.97 - 2.86	0.062
10-12	0.87	0.55 - 1.38	0.871	1.28	0.76 - 2.15	0.357
≥ 13	1	-	-	1	-	-
Hookworm						
infected	1	-	-	1	-	-
no eggs detected	0.67	0.47 - 0.97	**0.032**	0.99	0.65 - 1.52	0.970
Other Helminthes*						
infected	1	-	-	1	-	-
no eggs detected	0.42	0.21 - 0.84	**0.014**	0.74	0.33 - 1.66	0.32
Treatment						
Baseline	4.69	3.39 – 6.51	**<0.001**	5.28	3..68 – 7.57	**<0.001**
3mths	1.90	1.43 – 2.52a	**<0.001**	2.02	1.48 – 2.77	**<0.001**
6mths	1.08	0.94 – 1.23	0.288	1.09	0.94 – 1.26	0.280
9mths	1	-	-	1	-	-
Community						
Jagbengbendo	0.74	0.48 - 1.14	0.175	0.70	0.45 - 1.11	0.131
Kojobone	0.75	0.47 - 1.17	0.204	1.13	0.68 - 1.83	0.656
Takumdo	0.52	0.34 - 0.81	**0.004**	0.65	0.41 - 1.04	0.069
Wiae	1	-	-	1	-	-

§ cOR = crude Odds Ratio; ∞95% CI (LL – UL) = 95% confidence interval, LL = lower limit, UL = upper limit; * Other STHs = other Helminths which represent *T. truchiura*, and *H. nana*. No participant was found positive with *A. lumbricoides* throughout the study. Univariate and multi-

variate analyses with anaemia status as the outcome variable were conducted using logistic regression in the context of the generalized estimating equations (GEE) model. Significant associations are in boldface.
