# Supplementary material for: The effectiveness of albendazole against hookworm infections and the impact of bi-annual treatment on anaemia and body mass index of school children in the Kpandai district of northern Ghana
Source: PLoS One. 2024 Mar 1;19(3):e0294977. doi: 10.1371/journal.pone.0294977 (PMC10906822; doi:10.1371/journal.pone.0294977)
Supplement: S7 Table — (PDF) [file pone.0294977.s007.pdf]

S7 Table: Associations of risk factor-treatment interactions over time with anaemia status

**Parameter**	**Anaemia status (anaemic vs normal)**					
	**cOR[§]**	**95% CI (LL-UL)[∞]**	**Wald's P-val**	**adjusted OR**	**95% CI (LL-UL)**	**Wald's P-val**
**Treatment * Gender**						
Male * Baseline	5.26	3.19 - 8.64	**<0.001**	3.57	0.29 - 43.35	0.318
Female * Baseline	5.85	3.71 - 9.22	**<0.001**	3.98	0.31 - 50.28	0.285
Male * 3 months	2.22	1.43 - 3.46	**<0.001**	3.19	0.27 - 37.82	0.357
Female * 3 months	2.28	1.56 - 3.35	**<0.001**	3.55	0.29 - 42.16	0.316
Male * 6 months	1.52	0.98 - 2.35	0.059	0.29	0.01 - 6.31	0.435
Female * 6 months	1.09	0.91 - 1.32	0.345	0.21	0.01 - 4.34	0.313
Male * 9 months	1.44	0.93 - 2.23	0.098	1.45	0.92 - 2.29	0.105
Female * 9 months	1	-	-	1	-	-
**Treatment * Age**						
≤ 6 * Baseline	23.14	9.22 - 58.04	**<0.001**	6.08	2.40 - 15.39	**<0.001**
7-9 * Baseline	9.31	4.14 - 20.93	**<0.001**	2.48	1.08 - 5.67	**0.032**
10-12 * Baseline	5.5	2.62 - 11.56	**<0.001**	1.48	0.69 - 3.16	0.307
≥ 13 * Baseline	3.89	2.00 - 7.55	**<0.001**	1	-	-
≤ 6 * 3 months	12.41	5.57 - 27.67	**<0.001**	5.76	2.64 - 12.55	**<0.001**
7-9 * 3 months	2.13	1.04 - 4.36	**0.038**	0.97	0.49 - 1.95	0.942
10-12 * 3 months	2.07	1.02 - 4.18	**0.044**	0.96	0.48 - 1.92	0.915
≥ 13 * 3 months	2.2	1.08 - 4.49	**0.03**	1	-	-
≤ 6 * 6 months	4.79	2.35 - 9.74	**<0.001**	5.56	2.68 - 12.55	**<0.001**
7-9 * 6 months	1.67	0.82 - 3.43	0.158	1.93	0.92 - 4.02	0.079
10-12 * 6 months	1.09	0.54 - 2.23	0.806	1.19	0.57 - 2.48	0.641
≥ 13 * 6 months	0.92	0.69 - 1.23	0.563	1	-	-
≤ 6 * 9 months	3.38	1.69 - 6.78	**0.001**	3.27	1.61 - 6.65	**0.001**
7-9 * 9 months	1.59	0.78 - 3.26	0.201	1.63	0.79 - 3.37	0.19
10-12 * 9 months	1.2	0.59 - 2.45	0.612	1.11	0.54 - 2.29	0.785
≥ 13 * 9 months	1	-	-	1	-	-

§ cOR = crude Odds Ratio; ∞95% CI (LL – UL) = 95% confidence interval, LL = lower limit, UL = upper limit; ⸸Other STHs = other Helminthes which represent *T. truchiura*, and *H. nana*. No participant was found positive with *A. lumbricoides* throughout the study. Univariate and multi-variate analyses of the effect of treatment-covariate interactions over time with anaemia status (the outcome variable) were conducted using logistic regression in the context of the generalized estimating equations (GEE) model. Significant associations are in boldface.
