# Supplementary material for: The effectiveness of albendazole against hookworm infections and the impact of bi-annual treatment on anaemia and body mass index of school children in the Kpandai district of northern Ghana
Source: PLoS One. 2024 Mar 1;19(3):e0294977. doi: 10.1371/journal.pone.0294977 (PMC10906822; doi:10.1371/journal.pone.0294977)
Supplement: S8 Table — (PDF) [file pone.0294977.s008.pdf]

S8 Table: Associations of risk factor-treatment interactions over time with anaemia status

Parameter	Anaemia status (anaemic vs normal)					
	cOR[§]	95% CI (LL-UL)[∞]	Wald's P-val	adjusted OR	95% CI (LL-UL)	Wald's P-val
**Treatment * Hookworm**						
Uninfected * Baseline	3.44	1.55 - 7.66	**0.002**	1.38	0.72 - 2.64	0.326
Infected * Baseline	2.79	1.13 - 6.84	**0.025**	1	-	-
Uninfected * 3 months	1.28	0.59 - 2.76	0.533	0.87	0.39 - 1.98	0.744
infected * 3 months	1.69	0.61 - 4.67	0.313	1	-	-
Uninfected * 6 months	0.76	0.37 - 1.58	0.46	1.59	0.61 - 4.13	0.339
infected * 6 months	0.58	0.21 - 1.60	0.292	1	-	-
Uninfected * 9 months	0.67	0.31 - 1.47	0.316	0.64	0.27 - 1.56	0.328
infected * 9 months	1	-	-	1	-	-
**Treatment * Other Helminthes**[ⱡ]						
Uninfected * Baseline	2.78	0,46 - 16.94	0.268	0.66	0.16 - 2.62	0.55
Infected * Baseline	5.11	0.57 - 45.87	0.145	1	-	-
Uninfected * 3 months	1.16	0.19 - 6.95	0.87	0.61	0.15 - 2.41	0.479
infected * 3 months	1.78	0.21 - 15.33	0.601	1	-	-
Uninfected * 6 months	0.66	0.11 - 3.95	0.651	2.01	0.24 - 17.18	0.523
infected * 6 months	0.67	0.05 - 9.47	0.765	1	-	-
Uninfected * 9 months	0.61	0.10 - 3.71	0.593	1.11	0.12 - 10.06	0.93
infected * 9 months	1	-	-	1	-	-

§ cOR = crude Odds Ratio; ∞95% CI (LL – UL) = 95% confidence interval, LL = lower limit, UL = upper limit; ⱡOther STHs = other Helminthes which represent *T. truchiura*, and *H. nana*. No participant was found positive with *A. lumbricoides* throughout the study. Univariate and multi-variate analyses of the effect of treatment-covariate interactions over time with anaemia status (the outcome variable) were conducted using logistic regression in the context of the generalized estimating equations (GEE) model. Significant associations are in boldface.
