# Supplementary material for: The effectiveness of albendazole against hookworm infections and the impact of bi-annual treatment on anaemia and body mass index of school children in the Kpandai district of northern Ghana
Source: PLoS One. 2024 Mar 1;19(3):e0294977. doi: 10.1371/journal.pone.0294977 (PMC10906822; doi:10.1371/journal.pone.0294977)
Supplement: S1 Fig — Map was developed by Elias K. Asuming-Brempong using the QGIS Girona version 3.0.3 (Boston, MA., USA). Shape files of the regions of Ghana were obtained online (URL: https://github.com/tierney/gis-sandbox/tree/master/data/GIS-Ghana/ghana.shapefiles). Also, GPS data were obtained from the field, using appropriate devices, and exported to Microsoft Office Excel version 2013, where conversions were made. The document was then exported to the QGIS Girona version 3.0.3 software as a delimited text file. (PDF) [file pone.0294977.s009.pdf]

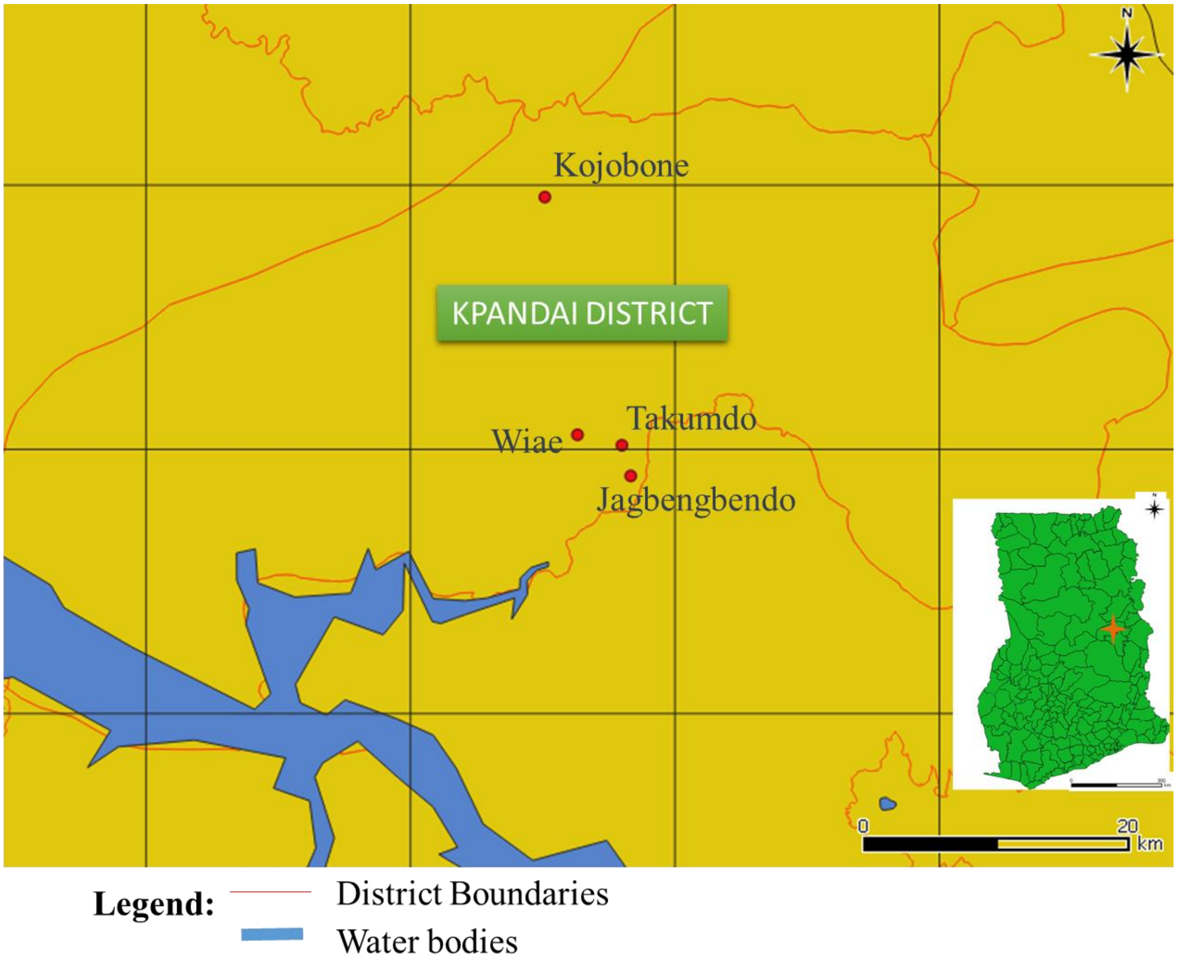

S1 Fig: A map of the Kpandai district indicating the locations of the study communities. Map was developed by Elias K. Asuming-Brempong using the QGIS Girona version 3.0.3 (Boston, MA., USA). Shape files of the regions of Ghana were obtained online (URL: https://github.com/tierney/gis-sandbox/tree/master/data/GIS-Ghana/ghana.shapefiles). Also, GPS data were obtained from the field, using appropriate devices, and exported to Microsoft Office Excel version 2013, where conversions were made. The document was then exported to the QGIS Girona version 3.0.3 software as a delimited text file.
