# Supplementary material for: The effectiveness of albendazole against hookworm infections and the impact of bi-annual treatment on anaemia and body mass index of school children in the Kpandai district of northern Ghana
Source: PLoS One. 2024 Mar 1;19(3):e0294977. doi: 10.1371/journal.pone.0294977 (PMC10906822; doi:10.1371/journal.pone.0294977)
Supplement: S2 Fig — Two ALB treatment interventions were administered to recruited SAC at baseline (after parasitological screening) and in the 6th month (after parameters of interest had been collected, and parasitological assessments done). Vertical, red-dashed lines demarcate treatment intervention time points. (PDF) [file pone.0294977.s010.pdf]

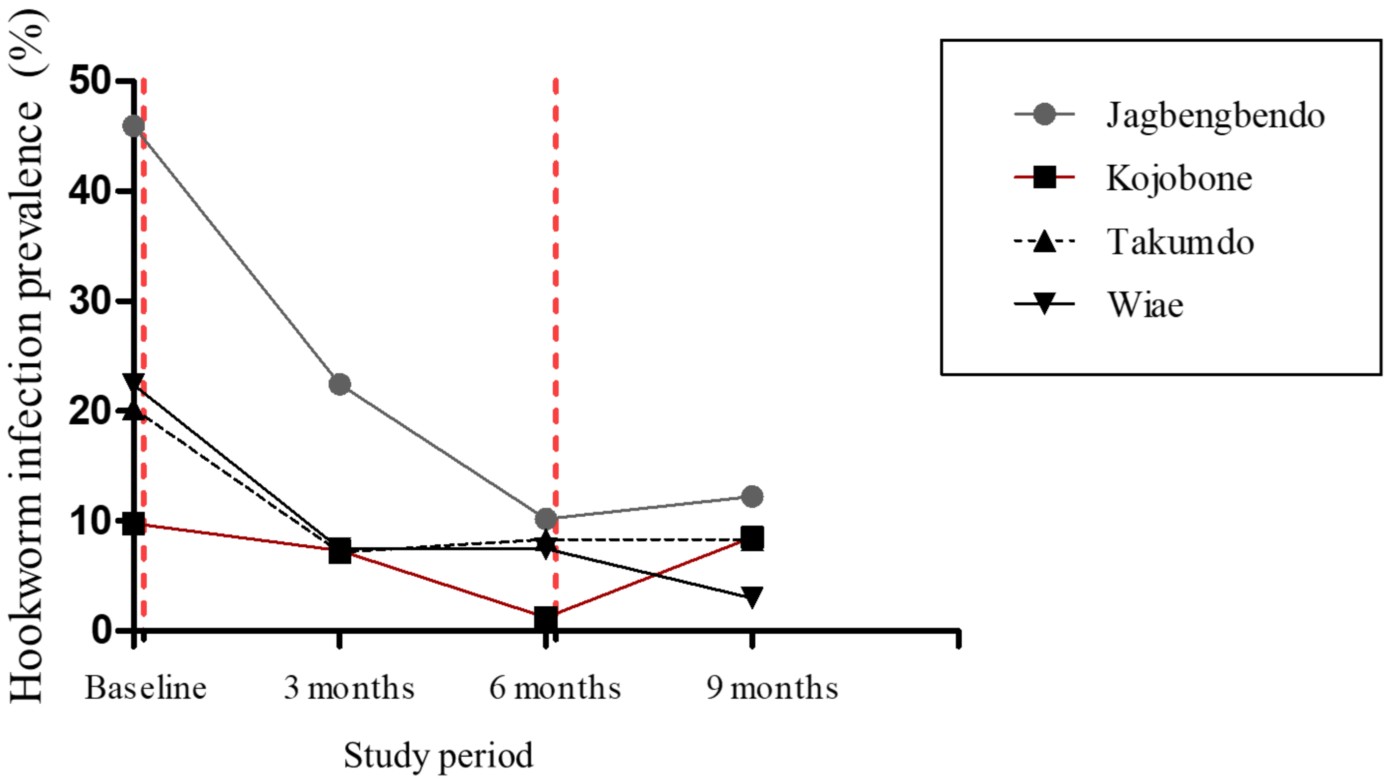

S2 Fig: Effect of Albendazole (ALB) treatment interventions on hookworm infection prevalences in the study communities over the study period. Two ALB treatment interventions were administered to recruited SAC at baseline (after parasitological screening) and in the 6th month (after parameters of interest had been collected, and parasitological assessments done). Vertical, red-dashed lines demarcate treatment intervention time points.
